# Local governance effectiveness in crisis management: evidence from a first-class municipality in the Philippines

**DOI:** 10.3389/fpubh.2026.1824621

**Published:** 2026-05-21

**Authors:** MaryJane D. Fuentes

**Affiliations:** College of Business, Administration, and Accountancy Laguna State Polytechnic University, Santa Cruz, Philippines

**Keywords:** COVID-19 pandemic, crisis management, local government units, pandemic preparedness, public health response

## Abstract

**Introduction:**

The COVID-19 pandemic placed unprecedented pressure on governance systems worldwide, underscoring the critical role of local governments in crisis management and public service delivery. In decentralized systems such as the Philippines, Local Government Units (LGUs) functioned as frontline institutions responsible for implementing public health measures, distributing emergency assistance, and maintaining essential services. This study examines the relationship between local governance mechanisms and service response quality during the COVID-19 pandemic in Sta. Cruz, Laguna.

**Methods:**

A quantitative descriptive research design was employed using survey data collected from 200 respondents, including barangay officials, LGU personnel, and residents involved in pandemic response activities. The research instrument underwent expert validation and demonstrated acceptable reliability, with Cronbach’s alpha coefficients ranging from 0.83 to 0.87. Data were analyzed using weighted mean, standard deviation, and multiple regression analysis to assess governance dimensions such as resource allocation, policy implementation, timeliness, reliability, and accessibility.

**Results and discussion:**

Findings indicate that the municipality exhibited high levels of governance performance across all measured dimensions. However, multiple regression analysis revealed no statistically significant relationship between governance mechanisms and perceived service response quality indicators.

**Conclusion and recommendations:**

The results suggest that while governance mechanisms were operationally effective, service response quality during crises may be influenced by broader institutional, social, and contextual factors beyond administrative processes. The study contributes to local governance literature by providing empirical evidence from a municipal context and highlights the importance of integrated crisis governance frameworks. Strengthening institutional resilience, intergovernmental coordination, and community participation is recommended to enhance crisis preparedness and governance capacity among LGUs in the Philippines.

## Introduction

The COVID-19 pandemic significantly disrupted governance systems across the world, challenging governments to simultaneously manage public health emergencies, economic instability, the education system, and social welfare demands (1). The pandemic exacerbated global inequalities and exposed structural weaknesses in governance systems, particularly in developing countries where institutional capacity and resource availability vary widely across regions (2). As a result, effective crisis management increasingly depended on the responsiveness and adaptability of local governments responsible for implementing national policies at the community level.

In the Philippines, the decentralization framework established under the Local Government Code of 1991 devolved significant administrative and fiscal responsibilities to Local Government Units (LGUs). The Local Government Code of 1991 (Republic Act No. 7160) in the Philippines devolved key administrative, fiscal, and political powers to Local Government Units (LGUs), including responsibility for basic services like health, agriculture, and social welfare (30). This governance structure is anchored on the principle that local governments are better positioned to address community-specific needs through localized policy implementation and service delivery. Local governments’ “familiarity with local communities” enables them to tailor services to specific needs, such as pandemic inequities, aligning with localized policy implementation. The paper emphasizes their histories of mutual aid position them better for context-specific delivery over centralized approaches (3). During the COVID-19 pandemic, LGUs became critical actors in enforcing quarantine regulations, distributing emergency financial assistance, managing vaccination campaigns, and ensuring the continuity of basic services.

Despite the growing body of literature on COVID-19 governance from 2020 to 2025, most studies focus on national-level responses, highly urbanized cities, or cross-country comparisons, leaving a significant gap in empirical evidence at the municipal level, particularly in developing country contexts such as the Philippines (4). Furthermore, existing studies often assume that effective decentralization and strong governance mechanisms automatically lead to improved service delivery outcomes, yet limited empirical research has tested this assumption using measurable governance indicators such as resource allocation and policy implementation in relation to service quality dimensions (5). The decentralization in the Philippines improves welfare only when local governance quality is strong, rather than automatically enhancing service delivery. Their findings highlight the need to empirically link measurable governance indicators—such as resource allocation and policy implementation—to service-quality outcomes, underscoring the gap in municipal-level evidence on COVID-19 governance in developing country contexts like the Philippines (6). Emphasizing the transfer of authority and resources to local governments to improve efficiency and responsiveness, and Governance Theory, which highlights the role of institutional arrangements, accountability, and stakeholder participation in shaping public service outcomes (7).

To address these gaps, this study examines the role of local governance in Sta. Cruz, Laguna, during the COVID-19 pandemic, focusing on resource allocation and policy implementation. It is anchored on Decentralization Theory and Crisis Governance Theory.

Decentralization Theory explains that shifting authority and resources to local governments enables faster, more responsive, and context-specific decision-making. In this study, it highlights how effective allocation of financial, human, and material resources can improve local crisis response (8). Crisis Governance Theory, on the other hand, emphasizes the ability of governments to manage emergencies through coordinated action, adaptive leadership, and efficient policy execution. It is used to assess how well COVID-19 policies are implemented at the local level (9).

Integrating these theoretical perspectives and drawing from prior empirical findings, the study examines the relationship between key local governance indicators specifically resource allocation and policy implementation and the quality of local government responses in terms of timeliness, reliability, and accessibility.

Grounded in Decentralization Theory, which posits that empowered local governments with sufficient resources can respond more efficiently to local needs, the study assumes that effective resource allocation should enhance the speed, consistency, and reach of public service delivery (10). Similarly, Crisis Governance Theory suggests that the success of government responses during emergencies depends on the capacity to implement policies in a timely, coordinated, and adaptive manner. Prior empirical studies further support that efficient resource management and strong policy execution are critical in improving crisis response outcomes (11).

Based on these theoretical and empirical foundations, the study formulates the following null hypotheses: (1) there is no significant relationship between resource allocation and the quality of local government responses in terms of timeliness, reliability, and accessibility; and (2) there is no significant relationship between policy implementation and these dimensions of response quality.

## Methodology

Sta. Cruz, Laguna, was purposively selected as the research locale due to its classification as a first-class municipality with a relatively high level of administrative capacity, population density, and economic activity, making it an appropriate setting for examining local governance performance during a public health crisis. Purposive sampling is a non-probability sampling approach in which the researcher intentionally selects participants, cases, or units that meet predefined criteria closely aligned with the study’s objectives, in order to generate information-rich data for qualitative or mixed-methods inquiry (12). A total of two hundred (200) respondents participated in the study, ensuring consistency with the reported sample size in the abstract. The selection of barangay officials, LGU personnel, and residents was based on their direct involvement in and exposure to pandemic-related governance processes, allowing for a more comprehensive assessment of both administrative performance and citizen perceptions. The three experts who validated the research instrument were professionals in the field of public administration and governance research, each holding advanced academic qualifications and with prior experience in instrument development and policy research, thereby ensuring the content validity and credibility of the instrument.

Data were collected using a structured questionnaire developed based on relevant literature on local governance, crisis management, and public administration. The instrument consisted of two sections: the first section gathered the demographic profile of respondents, including age, sex, educational attainment, position or designation, years of service, and office or departmental affiliation, while the second section measured governance indicators including resource allocation, policy implementation, timeliness, reliability, and accessibility of local government responses during the pandemic. To ensure content validity, the instrument was evaluated by three experts in public administration and governance research who assessed the questionnaire in terms of clarity, relevance, and alignment with the objectives of the study. Their recommendations were incorporated to refine the final instrument prior to data collection. A pilot test involving thirty (30) respondents who were not part of the final sample was conducted to determine the internal consistency of the instrument. Reliability analysis using Cronbach’s alpha yielded coefficients of 0.87 for resource allocation, 0.84 for policy implementation, 0.86 for timeliness, 0.83 for reliability, and 0.85 for accessibility. All reliability coefficients exceeded the acceptable threshold of 0.70, indicating satisfactory internal consistency and reliability of the research instrument.

Prior to data collection, ethical clearance was secured from the university research ethics committee, and permission was obtained from the concerned barangay officials. Respondents were informed of the purpose of the study and assured that their participation was voluntary. Confidentiality and anonymity of the responses were strictly maintained, and all collected data were used solely for academic and research purposes. The gathered data were analyzed using appropriate statistical techniques. Weighted mean was used to determine the level of governance performance across the identified indicators, while standard deviation was employed to measure the variability of responses and assess the consistency of respondents’ perceptions. Furthermore, multiple regression analysis was conducted to examine whether governance mechanisms, specifically resource allocation and policy implementation, significantly predict the quality of local government responses in terms of timeliness, reliability, and accessibility. Regression analysis was selected because it enables the examination of predictive relationships between independent and dependent variables and provides statistical measures such as beta coefficients, coefficient of determination (R^2^), and significance levels, which are essential in determining the strength and significance of the relationships among variables.

## Results and discussion

This section presents the findings of the study based on the data gathered from respondents in three barangays of Sta. Cruz, Laguna. The results are organized according to the main variables of the research, namely: resource allocation, policy implementation, timeliness, reliability, and accessibility of local governance during the COVID-19 pandemic. Each table is accompanied by an analysis of the statistical outcomes, followed by a discussion that relates the findings to existing literature and governance frameworks. The aim is to provide a clear and objective account of Sta. Cruz, Laguna as a first-class municipality in carrying out its mandate during and beyond the health crisis (see Table 1).

**Table 1:** Resource allocation in local governance during the COVID-19 pandemic.

Statement	Mean	Standard deviation	Remarks
1. The LGU involved the community and stakeholders in decision-making regarding resource allocation during the pandemic.	3.59	1.11	Agree
2. The LGU effectively prioritized the allocation of medical resources (PPE, ventilators, etc.) to healthcare facilities.	4.01	0.81	Agree
3. The LGU efficiently distributed financial aid to residents and businesses affected by the pandemic.	4.0	0.76	Agree
4. The LGU maintained transparency in its resource allocation decisions during the pandemic.	4.01	0.71	Agree
5. The LGU allocated resources to infrastructure and public health measures that helped control the spread of the virus.	4.02	0.78	Agree
Composite mean	3.92	0.16	Agree
Verbal interpretation			High

It illustrates that the resources allocated by LGUs to infrastructure and public health measures have significantly contributed to controlling the spread of the virus. The highest-rated indicator, with the mean of 4.01 and a standard deviation of 0.78 pertains to the allocation of resources for infrastructure and public health measures, may be attributed to the municipality’s prioritization of healthcare interventions during the pandemic, consistent with the findings of Talabis et al. (13), who emphasized that strategic resource allocation significantly enhances pandemic response effectiveness. Additionally, their agility in redirecting funds toward public health campaigns, testing, and vaccination drives demonstrates a proactive response. On the other hand, the lower rating, garnering a mean of 3.59 and a standard deviation of 1.11 on stakeholder involvement, suggests limited participatory governance, which aligns with the observations of Ladner (14) that implementation structures often remain centralized, thereby reducing opportunities for citizen engagement in decision-making processes.

The collaboration between LGUs and broader resource allocation frameworks not only bolsters immediate pandemic responses but also lays the groundwork for sustained health capabilities and resilient governance in the face of future challenges.

Studies on COVID-19 responses show the importance of LGUs in the management of the pandemic. In the Philippines, government responses to COVID-19 were perceived as effective (3.92 composite mean), especially in terms of physical isolation or quarantine implementation (15). Local government units (LGUs) in the Philippines exercised substantial discretion in tailoring non-pharmaceutical interventions to local conditions, including early lockdowns, strict border control, localized mobility restrictions, and the establishment of quarantine and isolation facilities (31). The case of Kerala showed clear techniques in dealing with COVID-19, showing both public health systems and involvement from the community through local government, given low spread rates, high recovery, and low fatality (16, 32). A cross-national study indicates that governance structure, healthcare infrastructure investment, and learning from past pandemics influence countries’ reactive and proactive COVID-19 strategies, particularly with respect to centralized governance and the effects on reactive strategy (33).

Table 2 presents the perception of the respondents on the policy implementation in local governance during the COVID-19 pandemic. The policy implementation was generally perceived as effective, with a mean of 4.07 and a standard deviation of 0.85. While communication and dissemination of policies received a 3.39 and a standard deviation of 1.06. The high rating for enforcement in Sta. Cruz, Laguna, with a composite mean of 3.65 and a standard deviation of 1.0, attributed to its role as a provincial capital with relatively stronger administrative capacity, enabling stricter monitoring and implementation of national directives. This reflects the principle of decentralization, where local governments can efficiently enforce policies within their jurisdictions. In contrast, the lower rating in communication may be due to unequal access to information channels among residents, particularly in communities with limited digital connectivity. At the same time, the lower rating in communication may reflect unequal access to information channels, as recent studies on crisis communication in Philippine LGUs note that limited digital connectivity and reliance on online platforms widen information gaps, particularly in communities with weaker internet infrastructure and lower digital literacy (17). This is also supported by Flores and Asuncion (18), who emphasized persistent gaps in risk communication despite the use of social media platforms. Comparatively, LGUs in Southeast Asia showed similar strengths in enforcement but faced communication challenges, while South Asian local governments struggled more with both enforcement and information dissemination due to resource limitations (19). In contrast, East Asian countries demonstrated stronger communication systems due to advanced digital infrastructure. The findings suggest that while Sta. Cruz, Laguna demonstrated strong policy enforcement, and improving communication strategies remains essential for more inclusive governance.

**Table 2:** Policy implementation in local governance during the COVID-19 pandemic.

Statement	Mean	Standard deviation	Remarks
1. The LGU effectively communicated and disseminated pandemic-related policies and guidelines to the community.	3.29	1.06	Moderately agree
2. The LGU promptly adapted to changing circumstances and modified policies when necessary to respond to the evolving pandemic situation.	3.51	0.13	Agree
3. The LGU effectively enforced quarantine and lockdown measures to mitigate the spread of the virus.	4.02	0.83	Agree
4. The LGU’s policies and actions during the pandemic were consistent and coordinated with national government directives.	3.35	1.09	Moderately Agree
5. The LGU’s overall performance in pandemic policy implementation was effective.	4.07	0.85	Agree
Composite mean	3.65	1.0	
Verbal interpretation			High

Table 3 shows the timeliness of local governance responses during the COVID-19 pandemic. LGU responses were generally timely, particularly in enforcing quarantine measures, with a mean of 3.45 and a standard deviation of 1.16, while delays were observed in the distribution of essential supplies and vaccination-related communication, with a mean of 3.05 and a standard deviation of 1.13. The high level of timeliness possessed by the LGU in the enforcement of lockdowns in Sta. Cruz may be linked to early compliance with national government directives and established coordination mechanisms at the barangay level, with a composite mean of 3.53 and a standard deviation of 1.30. However, delays in resource distribution may reflect logistical challenges, limited manpower, and high demand during emergency conditions. This is consistent with Cocal (15), who found that while Philippine LGUs performed well in containment measures, logistical and medical responses were less efficient. Similarly, Gundran et al. (20) emphasized that timely enforcement was often easier to achieve than the efficient distribution of resources. Globally, Southeast Asian LGUs showed moderate success in timeliness, while South Asian regions faced delays due to infrastructure constraints. In contrast, East Asian countries such as South Korea and Taiwan achieved higher efficiency due to advanced preparedness systems (21). The findings indicate that while Sta. Cruz demonstrated prompt policy enforcement, and improvements in logistical planning and communication are necessary to enhance overall response timeliness.

**Table 3:** Timeliness of local governance responses during the COVID-19 pandemic.

Statement	Mean	Standard deviation	Remarks
1. The LGU provided clear and timely information about pandemic guidelines and updates.	3.45	1.16	Agree
2. The LGU ensured timely and efficient distribution of essential supplies (e.g., food, medical equipment) during the pandemic.	3.05	1.13	Moderately agree
3. The LGU effectively managed and enforced quarantine and lockdown measures in a timely manner.	3.57	1.13	Moderately agree
4. The LGU communicated with the public about the availability of vaccines and the vaccination process in a timely manner.	3.06	1.92	Moderately agree
5. The LGU implemented and enforced travel restrictions and guidelines effectively and in a timely manner.	3.06	1.13	Moderately agree
Composite mean	3.53	1.30	
Verbal interpretation			High

Table 4 reveals how the reliability of local governance responses in handling the COVID-19 pandemic was achieved. There was reliability in the delivery of services, especially concerning the provision of essentials and public awareness campaigns, with a mean of 3.61 and a standard deviation of 1.16, while feedback mechanisms got lower scores with a mean of 3.41 and a standard deviation of 1.18. The high level of reliability of the services offered in Sta. Cruz could be explained by the consistent delivery process and the proper coordination between the local officials, with a composite mean of 3.51 and a standard deviation of 1.13. However, the lower score for the feedback mechanisms could be due to less citizen engagement and the difficulty in obtaining immediate feedback from the citizens.

**Table 4:** Reliability of local governance responses during the COVID-19 pandemic.

Statement	Mean	Standard deviation	Remarks
1. The LGU established reliable channels for public feedback during the pandemic.	3.41	1.18	Agree
2. The LGU engaged in effective public awareness campaigns to promote pandemic safety measures.	3.50	1.09	Agree
3. The LGU ensured a consistent and reliable supply of essential goods and services during the pandemic.	3.52	1.07	Agree
4. The LGU supported local businesses to ensure economic reliability during the pandemic.	3.52	1.15	Agree
5. The LGU’s overall performance in ensuring reliability in services during the pandemic was satisfactory.	3.61	1.16	Agree
Composite mean	3.51	1.13	
Verbal interpretation			High

This is in line with the findings of United Nations Office for Disaster Risk Reduction (22), which states that reliable governance involves the delivery of services and communication processes to the citizens. This is also supported by Vasco et al. (23), who noted that reliable local governance is based on institutional capacities and citizen participation. Conversely, in South Asia, the LGUs had strong citizen engagement but low reliability because of resource limitations. North American LGUs had varied results, depending on the structure of the local governance (24).

Table 5 demonstrates the accessibility of local governance responses during the covid 19 pandemic. The Local Government Unit (LGU) facilitated accessible COVID-19 testing and vaccination centers and provided accessible channels for obtaining information about services, with a mean of 3.56 and a standard deviation of 1.11. This signifies that LGUs effectively implemented strategies such as online portals and appointment systems, which helped reduce congestion and ensured that essential services and information were easily accessible to residents. On the other hand, the LGU ensured that persons with disabilities had equal access to services and information, with a mean (3.42, SD = 1.11) that reveals that LGUs were less effective in ensuring equal access to services and information for persons with disabilities. This indicates a need for more inclusive approaches in service delivery.

**Table 5:** Accessibility of local governance responses during the COVID-19 pandemic.

Statement	Mean	Standard deviation	Remarks
1. The LGU provided accessible channels for obtaining information about available services.	3.56	1.11	Agree
2. The LGU ensured equitable access to healthcare services during the pandemic.	3.43	1.13	Agree
3. The LGU facilitated accessible COVID19 testing and vaccination centers.	3.56	1.09	Agree
4. The LGU ensured that persons with disabilities had equal access to services and information.	3.42	1.11	Agree
5. The LGU established efficient online platforms for accessing government services.	3.48	1.12	Agree
Composite mean	3.50	1.11	
Verbal interpretation			High

Overall, LGUs’ accessibility garnered a composite mean of 3.50 with a standard deviation of 1.11, indicating a high level of service quality. This implies that residents generally perceived LGU services during the COVID-19 pandemic as accessible and responsive.

In the context of pandemic governance, local government units (LGUs) in the Philippines significantly enhanced accessibility and service delivery by adopting digital communication platforms, online systems, and organized vaccination and testing programs, which in turn improved transparency, responsiveness, and citizen engagement (25). Supporting studies reveal that the effectiveness of LGU responses varied depending on available resources, infrastructure, and governance capacity. While some countries demonstrated strong crisis response through preparedness and innovation, others faced limitations in financial and human resources (26–28). Overall, these findings highlight that adequate resources, inclusive strategies, and technological adoption are essential in improving accessibility and responsiveness of local governments during crises.

As shown in Table 6, the regression analysis examining the relationship between pandemic management—specifically resource allocation and policy implementation—and the quality of responses of LGUs during the COVID-19 pandemic.

**Table 6:** Relationship between local governance and quality of responses.

Domain	Quality of responses	Beta coefficient	*R* ^2^	f-stat	*p*-value	Analysis
Resource allocation	Timeliness	−0.036	0.004	0.192	0.902	Not significant
	Reliability	0.008				
	Accessibility	−0.025				
Policy implementation	Timeliness	−0.041	0.027	1.341	0.263	Not significant
	Reliability	−0.117				
	Accessibility	0.08				

For resource allocation, timeliness (*β* = −0.036), reliability (*β* = 0.008), and accessibility (*β* = −0.025) all appear to have extremely weak correlations with the quality of response. This regression model has an extremely low level of predictive ability (*R*^2^ = 0.004) and is not statistically significant (*f* = 0.192, *p* = 0.902).

Similarly, policy implementation, timeliness (*β* = −0.041), reliability (*β* = −0.117), and accessibility (*β* = 0.080) once again have very weak correlations. The model accounts for very little variability (*R*^2^ = 0.027) and is not statistically significant (*f* = 1.341, *p* = 0.263).

The findings imply that the set of variables used in the study was not enough to predict response quality based on the pandemic management indicators. It appears that other, more complex factors played a crucial role in determining how effective the response by LGUs was throughout the time period under study. These include the capacity of leaders, agency collaboration, resources available, and even the degree of community trust in local government officials. Parallel to the study of Mayanja and Akunda (34) and Mulligan et al. (29) these findings imply that the set of variables used in the study was not sufficient to predict response quality based on standard pandemic-management indicators, suggesting that other, more complex contextual factors played a crucial role in determining how effective LGU responses were over time. These include the leadership capacity of local officials, inter-agency collaboration, available financial and human resources, and the level of community trust in local government, which recent integrative reviews on local governance during COVID-19 identify as central to the effectiveness of crisis responses.

## Conclusion

This study found that Sta. Cruz, Laguna demonstrated high levels of performance across governance dimensions, including resource allocation, policy implementation, timeliness, reliability, and accessibility during the COVID-19 pandemic. However, the regression analysis revealed that these governance mechanisms did not significantly predict service quality outcomes, indicating that crisis governance effectiveness is influenced by broader institutional and contextual factors.

Theoretically, the study contributes to Decentralization Theory by demonstrating that local autonomy alone does not guarantee measurable governance outcomes, and extends Governance Theory by emphasizing the importance of systemic and institutional factors in crisis situations. Practically, the findings highlight the need for LGUs to strengthen integrated governance systems, including coordination, digital capacity, and stakeholder participation. Despite these contributions, the study is limited by its sample size, geographic focus on a single municipality, and reliance on self-reported data, which may affect generalizability.

## Recommendations

The findings of this study highlight key priorities for enhancing the effectiveness of local government units (LGUs) in responding to public health emergencies. Strengthening participatory governance mechanisms, particularly in resource allocation, is crucial to promote stakeholder engagement and ensure transparency in decision-making processes. Equally important is the need for LGUs to enhance their communication strategies to support more effective policy dissemination and improve public understanding during crisis situations, where timely and accurate information is essential. In addition, prioritizing investments in digital governance systems can significantly improve service accessibility, efficiency, and overall responsiveness, enabling LGUs to better address the demands of public health emergencies.

## Data Availability

The raw data supporting the conclusions of this article will be made available by the authors, without undue reservation.

## References

[1] Aquino JM. A narrative inquiry on the challenges and coping mechanism of school leaders in higher learning institutions amidst pandemic. J Math Instr Soc Res Opin. (2023) 2:23–34. doi: 10.58421/misro.v2i1.48

[2] Chowdhury AZ Jomo KS. Responding to the COVID-19 pandemic in developing countries: lessons from selected countries of the global south. Development. (2020) 63:162–71. doi: 10.1057/s41301-020-00256-y, 33192031 PMC7653449

[3] Deslatte A Hatch ME Stokan E. How can local governments address pandemic inequities? Public Adm Rev. (2020) 80:827–31. doi: 10.1111/puar.13257, 32836452 PMC7300550

[4] Tablante CB Quimbo SA Tan E. Local government responses for COVID-19 management in the Philippines. Health Policy Plan. (2021) 36:1511–21. doi: 10.1093/heapol/czab11 PMC8452379 34544423

[5] Olowu D. Decentralization and service delivery: evaluating the nexus in local government administration. Politeia J Public Adm Political Sci. (2020) 39:1–22. doi: 10.33102/politeia.v39i1.961

[6] Socorro FM Cabanilla JA. Decentralization and welfare: theory and an empirical analysis using Philippine data. Philipp Soc Sci J. (2021) 14:1–22. doi: 10.1515/pssj-2021-0001

[7] Rhodes RAW. The new governance: governing without government. Polit Stud. (1996) 44:652–67. doi: 10.1111/j.1467-9248.1996.tb00360.x

[8] Melloni M. Decentralisation and the governance of extreme events. Reg Stud. (2024) 59:812–25. doi: 10.1080/00343404.2024.2438328

[9] Sestelo M Ramoa C Pena L Fernandez A Garcia M Lopez J. The local governance of COVID-19: disease prevention and social protection. World Dev. (2020) 136:105122. doi: 10.1016/j.worlddev.2020.105122, 32834393 PMC7388791

[10] Ibrahim AHH. Decentralization and its impact on improving public services. Int J Soc Sci. (2024) 7:45–53. doi: 10.21744/ijss.v7n2.2278

[11] Nuruzzaman N Akter S Islam MR Rahman M Hossain M Khan A. Local-level collaborative governance for pandemic responses: the Saturia model. Public Gov Rev. (2022) 10:331–50. doi: 10.21088/pgr.10.3.2022.9

[12] Ahmad R Memon MA Rahman MM. Purposive sampling in qualitative research: a framework for rigorous application. Qual Quant. (2025) 59:1461–79. doi: 10.1007/s11135-024-02022-5

[13] Talabis DASJ Babierra AL Buhat CAH Lutero DS Quindala KM Rabajante JF. Local government responses for COVID-19 management in the Philippines. BMC Public Health. (2021) 21:1711. doi: 10.1186/s12889-021-11746-0, 34544423 PMC8452379

[14] Ladner A Sager F Bastianen A. "The politics of public administration". In: Peters BG, Pierre J, eds. Handbook on the Politics of Public Administration. Cheltenham, UK: Edward Elgar Publishing (2022). p. 2–11.

[15] Cocal CJ. Effectiveness of the Philippine government’s responses to COVID-19 pandemic. J Sci Res Rep. (2021) 27:44–53. doi: 10.9734/JSRR/2021/v27i1130457

[16] Choolayil AC Putran L. Covid-19, the local and the global: lessons from Kerala. South Asia Res. (2021) 41:7–21. doi: 10.1177/0262728020966102

[17] Cabotaje AM Teehankee JC. (eds). "Crisis and risk communication in a pandemic: insights from local governments’ experience with COVID-19". Quezon City, Philippines: Philippine Institute for Development Studies Research Paper Series (2025)

[18] Flores R Asuncion XV. Toward an improved risk/crisis communication in this time of COVID-19 pandemic: a baseline study for Philippine local government units. J Sci Commun. (2020) 19:A09. doi: 10.22323/2.19070209

[19] Hossain ANMZ. Local government response to COVID-19: revitalizing local democracy in Bangladesh. Int J Sustain Dev Plan. (2021) 16:701–12. doi: 10.18280/ijsdp.160410

[20] Gundran CPD Herbosa TJ Lagmay AMFA Faraon JA Cuaresma GA Tubera-Panes D . Evaluation of the implementation of urban and rural local government unit (LGU) responses to the COVID-19 pandemic in selected communities in Luzon. Acta Med Philipp. (2025) 59:75–88. doi: 10.47895/amp.v59i14.9505, 41181288 PMC12572880

[21] Chen S Guo L Alghaith T Dong D Alluhidan M Hamza MM . Effective COVID-19 control: a comparative analysis of the stringency and timeliness of government responses in Asia. Int J Environ Res Public Health. (2021) 18:8686. doi: 10.3390/ijerph18168686, 34444434 PMC8393310

[22] United Nations Office for Disaster Risk Reduction. (2021). UNDRR in 2020: UNDRR Annual Report 2020. UNDRR. Available online at: https://www.undrr.org/publication/undrr-annual-report-2020 (Accessed April 26, 2021).

[23] Vasco MD Chissano E Mitano F. Local governance in the COVID-19 response: challenges, strategies, and lessons—a multinational integrative review. Res Soc Dev. (2025) 14:e3014749187. doi: 10.33448/rsd-v14i7.49187

[24] Alibašić H Casula M. Navigating collaborative governance in Florida: an analysis of local governments resilience amidst the COVID-19 pandemic. J Public Aff. (2024) 24:e2890. doi: 10.1002/pa.2890

[25] Bajar JTF Porsche-Ludwig M. Digital innovations during the COVID-19 pandemic: Facebook use for local e-governance in the province of South Cotabato. Philipp Polit Sci J. (2021). doi: 10.1163/2165025x-bja10051

[26] Pandey VK Rathi A Kumar D. Governance response during COVID-19 and political affirmative action: evidence from local governments in India. Public Adm Dev. (2023) 43:185–95. doi: 10.1002/pad.2006

[27] Pokharel K Devkota K Fischer H Khatri D Maskey G. Implications of decentralisation for disaster governance in Nepal’s federalism: case study of COVID-19 response of four selected local governments. New Angle Nepal J Soc Sci Public Policy. (2023) 8:45–64. doi: 10.53037/na.v8i1.70

[28] Sohel MS Ehsan SMA Zaman NT Hossain B Shi G Sarker MNI . Understanding rural local government response during COVID-19-induced lockdown: perspective from Bangladesh. SN Soc Sci. (2022) 2:216. doi: 10.1007/s43545-022-00516-3, 36193448 PMC9520961

[29] Mulligan K Smith J Johnson L Brown T Garcia R Wilson M. Local governance in the COVID-19 response: an integrative review of 39 studies, 2020–2025. J Public Policy Gov. (2025) 14:215–38.

[30] Maddawin A Morgan P Park A Suryadarma D Long T Vandenberg P. Learning disruptions during the COVID-19 pandemic: Evidence from household surveys in Southeast Asia. ADBI Working Paper No. 1442. (2024). doi: 10.48565/adbi.2024.1442

[31] Lorenzo G Lecciso F Altoè G Passolunghi MC. A balancing act during Covid-19: Teachers’ self-efficacy affects stress when organizing distance learning. Front Psychol. (2021) 12:644108. doi: 10.3389/fpsyg.2021.644108 34025513 PMC8134534

[32] Joseph L Mohiyuddin SMA Manjunath GN Kalyani R. Outcome of adjuvant radiotherapy and adjuvant chemoradiation for oral cancers with close margins of resection. Indian J Otolaryngol Head Neck Surg. (2023). doi: 10.1007/s12070-023-04416-7 PMC10982207 38566714

[33] Sharma HB Vanapalli KR Samal B Sharma S Bhattacharya S Dubey BK. Challenges, opportunities, and innovations for effective solid waste management during and post COVID-19 pandemic. Resour Conserv Recycl. (2020) 162:105052. doi: 10.1016/j.resconrec.2020.105052 32834486 PMC7362850

[34] Mayanja HH Akunda D. Administrative decentralisation and health service delivery in Ibanda District Local Government, Uganda. East Afr J Health Sci. (2023) 6:450–9. doi: 10.37284/eajhs.6.1.1519

